# Purines for Rapid Identification of Stroke Mimics (PRISM): study protocol for a diagnostic accuracy study

**DOI:** 10.1186/s41512-021-00098-3

**Published:** 2021-05-20

**Authors:** Lisa Shaw, Sara Graziadio, Clare Lendrem, Nicholas Dale, Gary A. Ford, Christine Roffe, Craig J. Smith, Philip M. White, Christopher I. Price

**Affiliations:** 1 Stroke Research Group, Population Health Sciences Institute, Newcastle University, Henry Wellcome Building, Newcastle Upon Tyne, NE2 4HH UK; 2 NIHR Newcastle In Vitro Diagnostics Co-operative, Newcastle upon Tyne Hospitals NHS Foundation Trust, Royal Victoria Infirmary, Queen Victoria Road, Newcastle upon Tyne, NE1 4LP UK; 3 NIHR Newcastle In Vitro Diagnostics Co-operative, Translational and Clinical Research Institute, Newcastle University, William Leech Building, Newcastle Upon Tyne, NE2 4HH UK; 4 School of Life Sciences, Gibbet Hill Campus, The University of Warwick, Coventry, CV4 7AL UK; 5 Medical Sciences Division, University of Oxford, Level 3, John Radcliffe Hospital, Oxford, OX3 9DU UK; 6 Oxford University Hospitals NHS Foundation Trust, John Radcliffe Hospital, Oxford, OX3 9DU UK; 7 School of Medicine, Keele University, Guy Hilton Research Centre, Thornburrow Drive, Hartshill, Stoke-on-Trent, ST4 7QB UK; 8 Division of Cardiovascular Sciences, Manchester University, Oxford Road, Manchester, M13 9PL UK; 9 Manchester Centre for Clinical Neurosciences, Manchester Academic Health Science Centre, Salford Royal NHS Foundation Trust, Salford, M6 8HD UK

**Keywords:** Stroke, Mimic, Purine, SMARTChip, Diagnostic accuracy study

## Abstract

**Background:**

Rapid treatment of stroke improves outcomes, but accurate early recognition can be challenging. Between 20 and 40% of patients suspected to have stroke by ambulance and emergency department staff later receive a non-stroke ‘mimic’ diagnosis after stroke specialist investigation. This early diagnostic uncertainty results in displacement of mimic patients from more appropriate services, inappropriate demands on stroke specialist resources and delayed access to specialist therapies for stroke patients. Blood purine concentrations rise rapidly during hypoxic tissue injury, which is a key mechanism of damage during acute stroke but is not typical in mimic conditions. A portable point of care fingerprick test has been developed to measure blood purine concentration which could be used to triage patients experiencing suspected stroke symptoms into those likely to have a non-stroke mimic condition and those likely to have true stroke. This study is evaluating test performance for identification of stroke mimic conditions.

**Methods:**

Design: prospective observational cohort study

Setting: regional UK ambulance and acute stroke services

Participants: a convenience series of two populations will be tested: adults with a label of suspected stroke assigned (and tested) by attending ambulance personnel and adults with a label of suspected stroke assigned at hospital (who have not been tested by ambulance staff).

Index test: SMARTChip Purine assay

Reference standard tests: expert clinician opinion informed by brain imaging and/or other investigations will assign the following diagnoses which constitute the suspected stroke population: ischaemic stroke, haemorrhagic stroke, TIA and stroke mimic conditions.

Sample size: ambulance population (powered for mimic sensitivity) 935 participants; hospital population (powered for mimic specificity) 377 participants.

Analyses: area under the receiver operating curve (ROC) and optimal sensitivity, specificity, and negative and positive predictive values for identification of mimic conditions. Optimal threshold for the ambulance population will maximise sensitivity, minimum 80%, and aim to keep specificity above 70%. Optimal threshold for the hospital population will maximise specificity, minimum 80%, and aim to keep sensitivity above 70%.

**Discussion:**

The results from this study will determine how accurately the SMARTChip purine assay test can identify stroke mimic conditions within the suspected stroke population. If acceptable performance is confirmed, deployment of the test in ambulances or emergency departments could enable more appropriate direction of patients to stroke or non-stroke services.

**Trial registration:**

Registered with ISRCTN (identifier: ISRCTN22323981) on 13/02/2019 http://www.isrctn.com/ISRCTN22323981

## Background

In the UK, stroke is the third leading cause of death and the single largest cause of adult disability with an overall economic impact of approximately £9 billion per year [1]. The cause of stroke is either cerebral ischaemia (85%) or haemorrhage (15%), and evidence of cost-effective disability reduction exists for early access to specialist services (all patients: NNT 20) [2], intravenous thrombolysis (IVT) for ischaemic stroke <4.5 h since onset (10–15% patients: mean NNT 7) [3, 4] and intra-arterial mechanical thrombectomy (MT) for large vessel occlusion stroke (LVO) < 6h since onset (5–10% patients: NNT 3) [5]. To improve access to treatments, national policy is driving the creation of regional neuroscience centres (also called comprehensive stroke centres) with thrombectomy capability or hyperacute stroke units (HASU; also known as primary stroke centres) which deliver thrombolysis and provide stroke unit care [6]. Increasing numbers of patients with suspected stroke are being redirected past the nearest hospital in order to access specialist care. Although outcomes are best when stroke patients are treated rapidly in a HASU or regional neuroscience centre, the emergency care pathway for admission is inefficient because accurate initial diagnosis is often difficult.

Ambulance practitioners, as the first point of contact in the clinical pathway for the majority of suspected stroke admissions, use a validated assessment to identify stroke symptoms and make a decision about where to take the patient [7]. Clinical guidelines specify that any suspected stroke should be conveyed directly to the nearest specialist stroke care centre [7]. The most widely used assessment is the Face Arm Speech Test (FAST), which records facial weakness, arm weakness and/or speech disturbance [8]. Although FAST has good sensitivity, poorer specificity results in 30–50% of ‘FAST positive’ patients later receiving a non-stroke ‘mimic’ diagnosis (i.e. false positives) [8, 9]. At hospital emergency departments, emergency medical staff can quickly identify more non-stroke mimic conditions during clinical assessment than ambulance practitioners, but the mimic rate is still 20–30% [10] resulting in frequent referral of mimics to stroke units rather than to appropriate alternative services according to the underlying condition.

This early diagnostic uncertainty can therefore result in displacement of mimic patients from more appropriate local hospitals or medical specialties within a hospital, inappropriate demands on finite stroke specialist resources and delayed access to specialist care and time-critical reperfusion therapies for true stroke patients. For example, national audit in England consistently shows that 44% of confirmed stroke patients are not admitted to a HASU within 4 h of hospital arrival [11], and as it has been observed that 15–20% HASU beds are occupied by patients with mimic conditions for a typical stay of 2.5 days [12], one explanation for the inability to achieve stroke service standards is the impact of ‘false positive’ stroke admissions (i.e. mimics) upon finite specialist resources.

Blood biomarkers have potential advantages in emergency assessment but have not previously proven useful during the critical early stages of stroke as release occurs hours after onset, and complex assays are required [13–15]. However, evidence is now accumulating that detection of whole blood purine concentration (WBPC) may be able to assist with stroke versus mimic identification [16–19]. Purines are short half-life natural by-products from energy-producing metabolic pathways which accumulate rapidly during hypoxic tissue injury [16]. Hypoxia is a main pathophysiological mechanism when stroke is caused by ischaemia (due to arterial occlusion) or haemorrhage (due to associated pressure effects and vasoconstriction), but for most common mimic conditions, significant tissue hypoxia is not involved (e.g. migraine). Whilst purines are not a tissue-specific biomarker and are elevated in other hypoxic states, cerebral tissue is a particularly rich source because of its high metabolic activity and oxygen sensitivity. In the clinical context of suspected stroke, a significant WBPC difference is expected between common mimic conditions (low WBPC values) and true stroke (higher WBPC values).

**Fig. 1:**
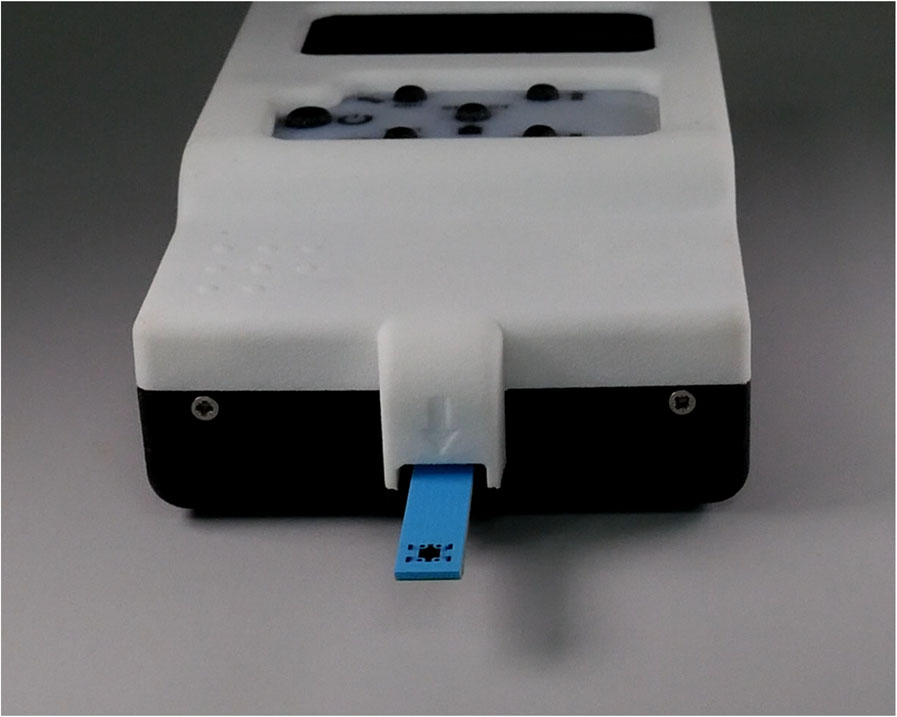
SMARTChip technology

A portable point of care novel biosensor assay ‘SMARTChip Purine’ has been developed to measure WBPC from a fingerprick drop of capillary blood [19]. The assay consists of enzymatic biosensors printed within a strip of carbon substrate and a bespoke reader device. A coupled cascade of three enzymes (adenosine deaminase, purine nucleoside phosphorylase and xanthine oxidase) quickly detects the combined concentrations of purine: adenosine, inosine and hypoxanthine [17]. Figure 1 shows the SMARTChip technology.

The SMARTChip assay could be used to triage patients with suspected stroke symptoms into those people likely to have a non-stroke mimic condition and those likely to have true stroke. The assay may have maximum impact if deployed pre-hospital but may also be of use on arrival at hospital for patients who do not present to the ambulance service, or if the technology is not available in the ambulance or was not deployed by ambulance practitioners because stroke was not suspected. In both settings, the assay result would be useful for determining whether patients with suspected stroke symptoms continue along the stroke emergency assessment pathway or whether they should be directed to other more appropriate emergency services for further assessment and treatment (i.e. the test result indicates that a patient is likely to have a non-stroke mimic condition).

The SMARTChip assay may also have an alternative purpose in the context of suspected stroke. Due to the greater volume of brain tissue involved during LVO stroke, it is hypothesised that WBPC readings will be elevated higher and for a longer time period when compared to both mimic conditions and those stroke patients without LVO. Sequential SMARTChip assay readings may therefore be able to assist in identification of patients with LVO stroke. As time-critical mechanical thrombectomy treatment is only available in a small number of regional neuroscience centres (comprehensive stroke centres), patients with LVO suitable for thrombectomy typically require secondary transfer from a HASU. Rapid identification of mechanical thrombectomy-eligible patients could facilitate transfer and expedite access to treatment.

The main aim of this study is to evaluate the diagnostic accuracy of the SMARTChip assay for identification of stroke mimic conditions amongst the suspected stroke population when used in either the pre-hospital or hospital environment. An exploratory substudy will be conducted to assess whether serial WBPC values could be useful for the identification of LVO stroke.

## Methods

### Study objectives

1. To determine the diagnostic accuracy of SMARTChip assay WBPC readings for identification of stroke mimic conditions when a reading is obtained in the pre-hospital setting, i.e. the test is conducted on patients suspected to have stroke by ambulance staff
2. To determine the diagnostic accuracy of SMARTChip assay WBPC readings for identification of stroke mimic conditions when a reading is obtained in hospital, i.e. the test is conducted on patients suspected to have stroke by hospital staff and when an ambulance test has not been undertaken
3. To develop pre-hospital and hospital statistical models which combine routinely available clinical data with SMARTChip assay WBPC readings to predict a stroke mimic diagnosis
4. To prospectively determine the diagnostic accuracy of the statistical models from objective 3
5. To report the failure rate of the SMARTChip assay when used in the pre-hospital and hospital settings

#### Substudy

1. To explore the diagnostic accuracy of two sequential SMARTChip assay WBPC readings for identification of LVO stroke using a reading obtained in the pre-hospital setting and a second reading obtained in the hospital setting
2. To develop and retrospectively explore the diagnostic accuracy of a statistical model which combines routinely available clinical data with pre-hospital- and hospital-obtained SMARTChip WBPC readings to predict the presence of LVO

### Study design

Two prospective blinded observational cohort studies will be conducted, one involving a convenience series of suspected stroke patients tested by hospital services and one involving a convenience series of suspected stroke patients tested by ambulance services. There will be 5 phases:

- Phase 1: pilot cohort study in hospital to review key technical performance parameters of the SMARTChip assay
- Phase 2: main hospital cohort study part 1. Once agreement is reached that technical performance is satisfactory, data will be collected to determine the diagnostic accuracy of WBPC assay readings for identification of mimic conditions (objective 2) and to build the statistical model (objective 3).
- Phase 3: main hospital cohort study part 2. Data will be prospectively collected to confirm the diagnostic accuracy of (i.e. validate) the hospital statistical model (objective 4).
- Phase 4: ambulance cohort study part 1. Data will be collected to determine the diagnostic accuracy of WBPC assay readings for identification of mimic conditions (objective 1) and to build the statistical model (objective 3).
- Phase 5: ambulance cohort study part 2. Data will be prospectively collected to confirm the diagnostic accuracy of (i.e. validate) the ambulance statistical model (objective 4).

Phases 4 and 5 may overlap with phases 2 and 3 depending on logistical factors, e.g. time taken to commence ambulance service involvement or availability of sufficient testing equipment.

Data collection to report the failure rate of the SMARTChip assay will run across phases 2–5. Data collection for the substudy is relevant to phases 4 and 5 only.

### Study setting

The hospital cohort study will take place within well-established acute stroke services with clinical access to CT or MR angiography (CTA/MRA) and at least daytime presence of a specialist stroke team. The ambulance cohort study will be hosted by regional ambulance services feeding into these stroke services. The research environment will reflect the local care pathway for patients with acute stroke symptoms including the scene of the incident, ambulance, emergency department and stroke service.

### Study participants

Both pre-hospital and hospital patients will fulfil the following criteria to undergo an assay reading:

Inclusion criteria:

- Aged 18 years and over
- At least responsive to strong stimuli during assessment of conscious level (alert, voice or pain on the Alert, Voice, Pain, Unresponsive (AVPU) scale)
- Face Arm Speech Test (FAST) positive or any observed new focal neurological symptoms indicating suspected acute stroke
- Persistence of the new stroke-like symptoms during the initial clinical assessment
- Believed to be within 6 h of onset of the new stroke-like symptoms at the time of the first clinical assessment
- SMARTChip assay WBPC reading can be undertaken before receipt of any reperfusion therapies
- Pre-hospital patients will only be included if they are to be transported to a study hospital
- Patients will only be included in the hospital cohort if they have *not* had a pre-hospital reading attempted

Exclusion criteria:

- Hypoglycaemia (capillary glucose <3.5mmol/l)
- External signs of significant acute trauma which are likely to need additional treatment (large haematomas, open wounds, limb deformity)
- Chemotherapy or radiotherapy treatment for cancer within the last 7 days

In order to provide a population to fulfil the objectives of the substudy, trained hospital staff (when available) will attempt an assay reading on the following subgroup of patients:

- Had an assay reading attempted by ambulance personnel
- The symptoms resulting in admission are believed to have commenced within 6 h of the time that the hospital assay can be performed
- The hospital assay can be performed before IVT or MT if this treatment is indicated

### Participant identification and consent

Ambulance and hospital personnel will determine suitability for the SMARTChip WBPC assay from their routinely conducted clinical assessments. Because the assessment of suspected stroke patients needs to be performed rapidly in order to minimise delays in accessing time-dependent treatments, patients will be approached about study enrolment after the initial emergency assessment and treatment processes, including the SMARTChip assay(s), have been completed. A formal research consent process performed in the ambulance or immediately on hospital arrival would cause unacceptable delays.

All patients who had either an ambulance and/or hospital WBPC reading attempted will be approached for study enrolment. An assay reading attempt will be defined as a fingerprick sample procedure being undertaken. As described below (see ‘SMARTChip Purine assay (index test)’), there may be occasions when the assay technology fails to calibrate and progression to fingerprick sampling is not possible. In such cases, patients will not have undergone any research procedures, and approach for consent will not occur. However, as failed calibration provides important test usability information, non-identifiable data about the test attempt will be recorded and reported.

Approach of patients for study enrolment will be by appropriately research-trained clinical staff or NHS research support staff. Ideally, approach of patients will take place during their inpatient stay and as soon as possible after the emergency assessments and treatments have taken place such that a timely discussion about the study can be held. However, for a small number of patients, this may not be possible because of early discharge, transfer or death, and in these situations, alterative consent methods will be used as described below.

#### Consent

The consent process will seek permission for retention and analysis of WBPC assay data and collection of selected routinely recorded healthcare data which are essential to complete the study objectives. There are no additional study-specific assessments.

### Consent for patients who can be approached about study participation during their inpatient stay

#### Consent for patients with mental capacity

For patients with capacity to consent to research, a trained member of the clinical team or NHS research support staff will approach the patient to discuss the study and provide a patient information sheet. After allowing sufficient time for potential participants to decide whether to take part in the study and an opportunity to ask questions, consent will be obtained in writing. When a patient has mental capacity but is unable to sign the consent form (e.g. because of weakness of the dominant hand following stroke), consent will be confirmed orally in the presence of a witness (an individual not otherwise involved in the trial), and the witness will sign and date the consent form on behalf of the participant.

If a potential participant is due to be discharged and wishes to have longer to consider the information before making a decision, staff will provide a postal consent form and prepaid reply envelope which can be returned if a decision to take part is made.

#### Consent for patients with mild communication difficulties

For patients with mild communication difficulties due to the effects of a stroke or mimic condition, a set of ‘easy access’ study documentation will be used. After allowing sufficient time for the information to be considered and an opportunity to ask questions, consent will be obtained in writing using the ‘easy access’ consent form.

If a potential participant is due to be discharged and wishes to have longer to consider the information before making a decision, an ‘easy access’ postal consent form and prepaid envelope are available for use. Staff will consider the appropriateness of such forms prior to issue including the availability of a relative/friend to assist with completion. If a postal form is judged to be inappropriate, the potential participant will be offered the opportunity to return for further discussion and consent at a later date.

#### Consent for patients who lack mental capacity

It is anticipated that approximately one third of study eligible patients will be unable to engage with an informed consent process due to the effects of stroke and mimic conditions upon communication and cognition. As exclusion of this group would drastically reduce the clinical relevance of the study, if a patient is unable to provide consent, a personal or nominated (professional) consultee will be approached as further described below.

It is anticipated that the majority of patients will be approached about participation within 24 h of admission which is typical for clinical trials of emergency stroke care. If at this time, a potential participant is believed to be lacking in capacity to consent to research, the staff making this first approach (appropriately trained clinical staff or NHS research support staff) will confer with the attending clinical team to determine the likelihood that this patient will improve and recover capacity by 48 h after admission. If it is considered that the patient is unlikely to recover capacity in this time, staff will proceed to attempt to identify an appropriate personal consultee (usually the next of kin) to approach, discuss the study and provide a consultee information sheet. If a personal consultee is identified, after allowing sufficient time for him/her to consider the patient’s wishes and feelings and an opportunity to ask questions, the consultee will be asked to complete a consultee declaration form if they believe that the patient would have no objection to taking part in the study. If the potential participant is due to be discharged and the personal consultee wishes to have longer to consider participation, staff will provide a postal personal consultee declaration form and prepaid reply envelope which can be returned if a decision to take part is made.

In the event of being unable to locate an appropriate personal consultee by 48 h after admission, an independent clinician (nominated consultee) will be approached to confirm that the patient lacks capacity for consent and that study participation would not introduce a risk of harm or be against the patient’s wishes from what is known about their character and beliefs. The independent clinician will sign a nominated consultee declaration form concerning study participation.

If when a patient is first approached they are believed to be lacking in capacity to consent to research and after conferring with the clinical team it is considered that the patient is improving and therefore may recover capacity to discuss the study, approach about consent will be delayed for 24 h. A further review of capacity will then be undertaken. If at this time the patient has recovered capacity, staff will proceed to seek consent directly from the patient. However, if the patient remains unwell and lacking in capacity, staff will proceed as described above to approach a personal consultee or independent clinician.

#### Consent and early mortality

The early mortality rate following acute stroke is approximately 10%. Some mimic conditions are also associated with a high mortality, e.g. severe infection. Exclusion of patients that die soon after admission would reduce the study’s relevance for the typical suspected stroke population.

If a patient who underwent a SMARTChip assay attempt dies before consent can be obtained using one of the approaches described above, the local principal investigator will sign an Early Mortality Declaration Form to confirm that the patient has died, and take responsibility for the use of routinely collected healthcare data for this research project.

##### Consent for patients who are only identified after discharge or transfer from the admitting hospital

Patients who are only identified as having undergone a SMARTChip assay after discharge or transfer from the admitting hospital will be invited to take part by post. An invitation letter, participant information sheet, consent form and prepaid return envelope will be mailed. The letter will include a telephone number of the admitting hospital research team to answer any queries or discuss the study in more detail. Patients willing to take part in the study will be asked to return a completed consent form.

As it will not be possible to assess mental capacity or communication issues prior to a postal invitation, the invitation letter includes a specific section for a person reading the letter who is not the intended recipient but reading it on their behalf. The reader is informed that the recipient can take part in the research and is asked to contact the hospital team for discussion and further information. If contacted, the hospital team will discuss the study and offer either a face-to-face appointment to obtain consent or to post the appropriate form (i.e. the easy access postal consent form or the personal consultee postal declaration form).

For invited patients who have not returned a consent form within 2 weeks, or where there has been no other contact about an invited patient, the local hospital research team will follow up with one telephone call.

##### Changes in capacity to consent to research during participation in the study

As there are no additional study-specific assessments and only collection of routinely available healthcare data in this project after the SMARTChip assay, changes in capacity status will not be reviewed.

##### Consent not obtained

If a patient or a consultee declines the invitation to be included in the study or a postal consent form is not returned, or if consent by one of the approaches above is not obtained for any other reason, collected data will be retained at the local site to document that a SMARTChip assay measurement(s) was undertaken but no further study data will be collected. The researchers will be informed that a test was conducted but consent was not obtained and no further data will be provided.

**Fig. 2:**
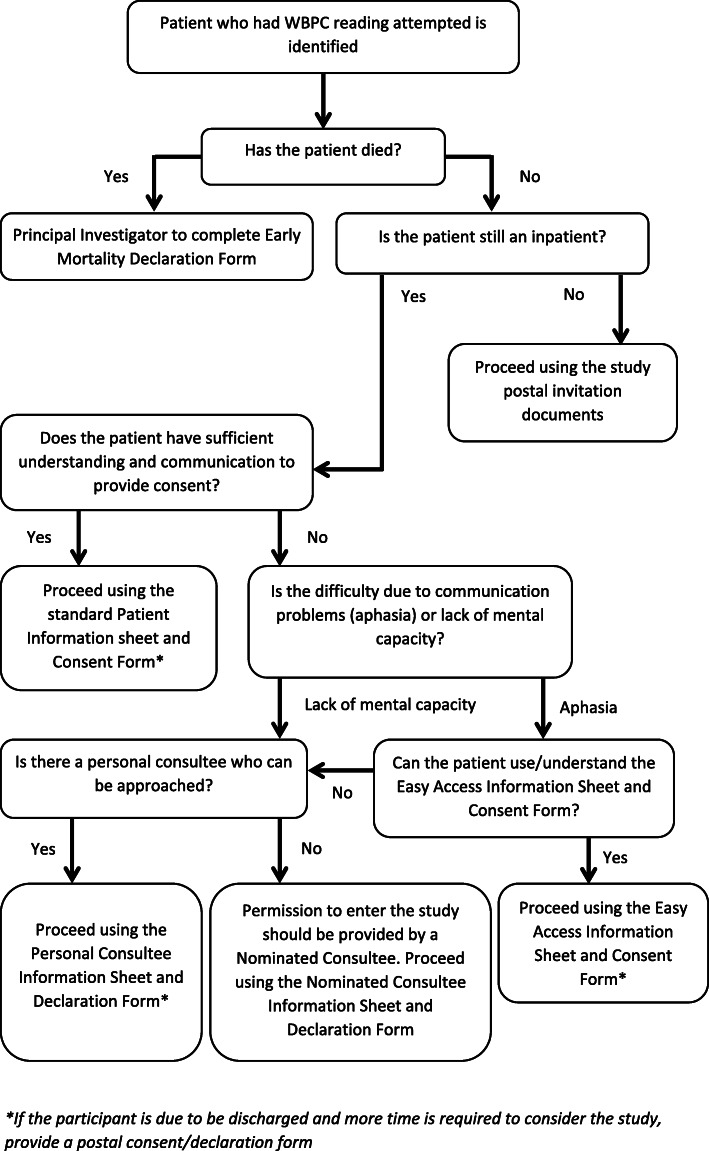
Decision process for study consent

Figure 2 summarises the decision process for obtaining study consent.

### SMARTChip purine assay (index test)

The diagnostic technology under evaluation comprises four tiny electrochemical biosensors printed in carbon on strip of ceramic substrate (50 × 10 × 1 mm) (called ‘SMARTChip Purine’) and a bespoke portable reader device. A coupled cascade of three enzymes (adenosine deaminase, purine nucleoside phosphorylase and xanthine oxidase) detects the combined concentrations of the purines: adenosine, inosine and hypoxanthine. To take a measurement, the user inserts the SMARTChip into the reader, performs a calibration and buffer step and then adds a drop of blood from a fingerprick sample. The procedure takes 6 to 8 min and can be completed in parallel with other aspects of the standard emergency care pathway for suspected stroke. As the study is blinded, the assay reading is not displayed anywhere and is only accessible to the researchers in the data downloaded from the reader (see below).

There may be occasions when an assay step fails, i.e. calibration, buffer or the WBPC measurement. If any step fails, to repeat the assay, a new SMARTChip is required. In the pre-hospital setting, only one attempt using one SMARTChip will be permitted to avoid the possibility of causing delays to patient transport to hospital, e.g. if calibration fails, the procedure will be abandoned at this point. For the hospital setting, if the calibration or buffer step fails, up to two further attempts will be permitted as this is unlikely to delay care (i.e. use up to three SMARTChips). However, only one fingerprick will be permitted and therefore only one attempt at the blood measurement step.

Following any assay attempt, the bespoke reader device will be connected to a designated password-protected study laptop, and reading data will be downloaded. Data will subsequently be provided to the research team (consented patients only) either manually via encrypted USB drives or over the internet. No patient identifiable data is added to the reader or downloaded into the study laptop.

#### Verification of WBPC assay results

Each WBPC assay result combines readings from the four individual SMARTChip electrodes. Due to the conditions under which SMARTChip will be deployed during the study, including handling by users who are less familiar with the technology, it is possible that the electrical contacts, electrodes or enzyme coating are damaged. Even though the SMARTChip passes the calibration stage, one or more of the four small electrodes may produce readings which are not physiologically or electrochemically plausible. The raw data from an assay passes through additional reader software to compute the WBPC result. This software will label the readings as electrochemically plausible ('verified') or not. All data will be used in the study analyses, but 'verified' and ‘unverified’ results will be handled separately (see ‘Main ambulance and hospital study analyses’ section).

In addition, staff from Sarissa Biomedical (technology manufacturer) will subject the biosensor current readings to an automated check using validated software. This is to review that the data quality controls within the reader are functioning appropriately. In the event that the automated check suggests a malfunction (for example a verified reading should be unverified or vice versa), a report will be prepared and presented to the study steering committee who will make a decision about whether there is justification to alter the reading accordingly. The automated check will occur without access to any clinical data, and any alterations will be recorded.

Unverified readings may be related to malfunctioning SMARTChips or user error, and this will be monitored by staff from Sarissa Biomedical. If there is a clustering of unverified readings associated with a particular production run of SMARTChips; then, others from that batch will be replaced and local storage conditions reviewed. If the assay data generated by any user shows a pattern suggestive of incorrect SMARTChip use, the user will be invited to attend refresher training.

### Reference standards (comparator)

#### Main study

For the main study, reference standards are required to assign the following clinical diagnoses which constitute the suspected stroke population: ischaemic stroke, haemorrhagic stroke, TIA and stroke mimic conditions. Whilst brain imaging tests are available which objectively confirm haemorrhagic stroke, no single diagnostic test exists for ischaemic stroke, TIA and many stroke mimic conditions. Because of this, diagnoses will be assigned by a local hospital expert clinician and confirmed via independent adjudication as described in detail below.

Hospital expert clinician opinion informed by brain imaging ± other investigations as clinically appropriate will be used to select a diagnosis from a predefined diagnosis framework (Table 1). This framework is being used because primary medical diagnoses recorded in medical records can vary according to the taxonomy used and the terminology preferred by individual clinicians, e.g. chest infection is synonymous with pneumonia, bronchopneumonia and lower respiratory tract infection. Clinicians will be asked to select a ‘definite’ or ‘probable’ primary clinical diagnosis according to the framework. As diagnoses are sometimes uncertain for a time after admission to hospital, clinicians will be asked to provide the diagnosis assigned at 7 days after hospital admission or at discharge/death if sooner. The framework includes an option for ‘unclear’ if the clinician cannot assign a diagnosis. In order to facilitate consistent completion of the framework, guidance has been developed to assist clinicians in allocation of a definite, probable or unclear diagnosis (Table 2).

**Table 1:**
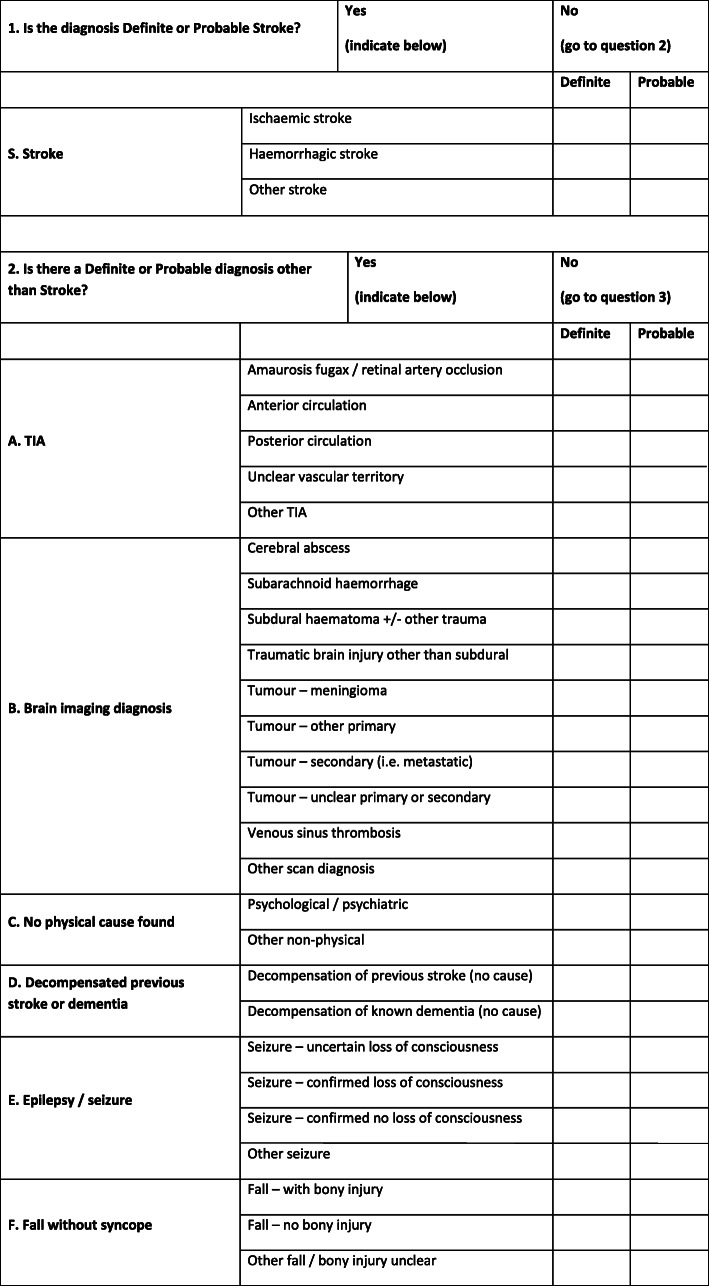

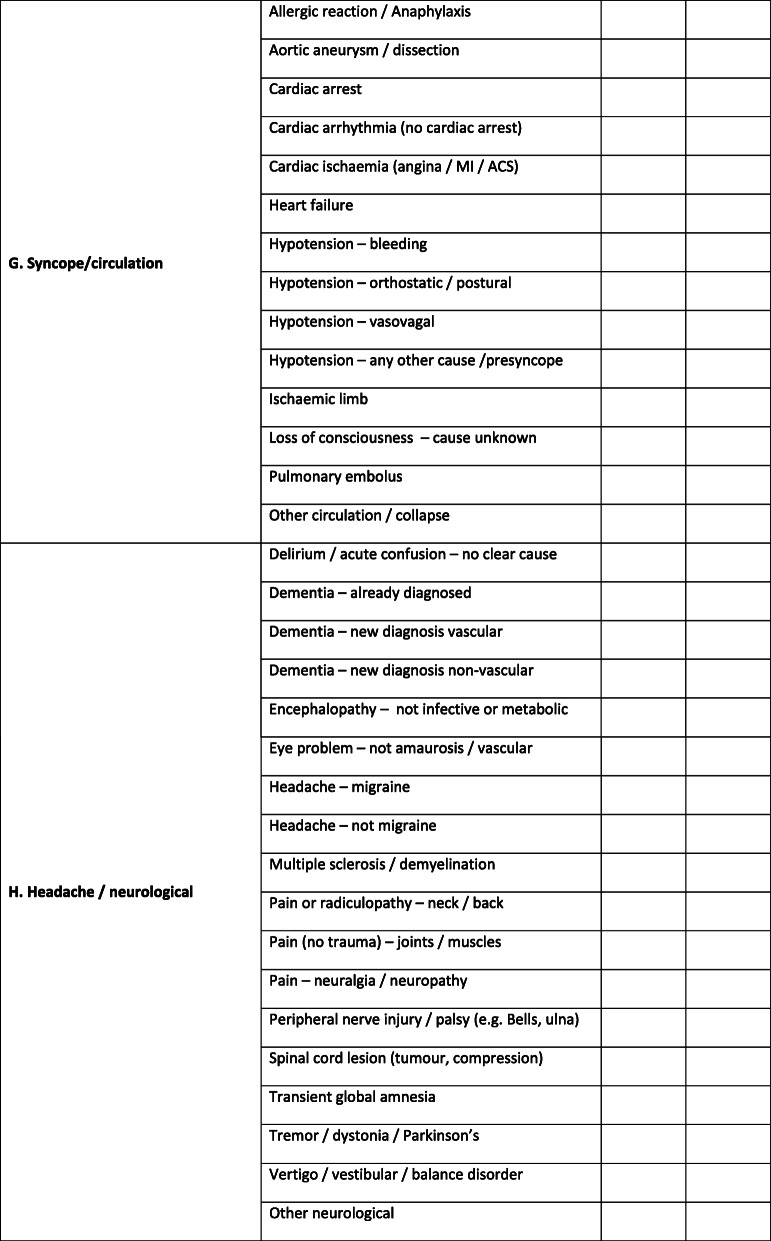

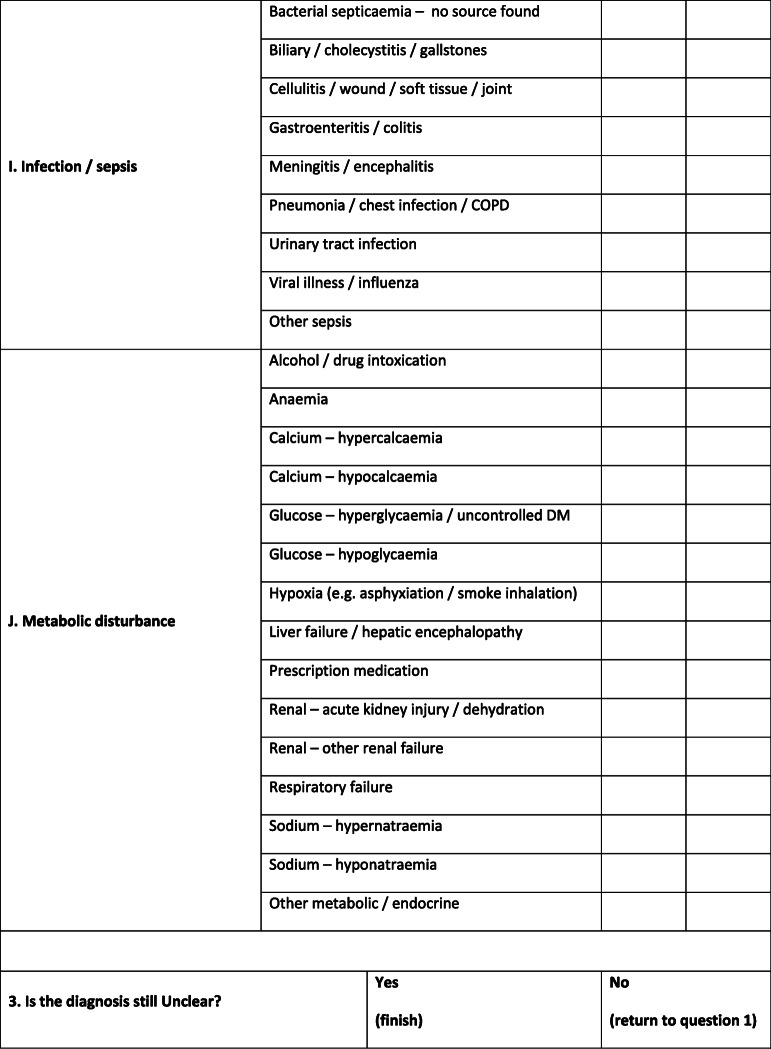
Primary diagnosis framework

**Table 2:** Guidance for completion of the primary diagnosis framework

Framework diagnosis	Clinical information may include	Investigations	Secondary prevention planned
**Definite stroke/TIA**	Symptoms typical for a vascular territory. Time course typical of stroke or TIA. Vascular risk factors are present.	Imaging supports the clinical diagnosis with no suggestion of an alternative aetiology.	Full (as appropriate)
**Probable stroke/TIA**	Symptoms mostly typical of a vascular territory. Time course suggestive of stroke or TIA. No vascular risk factors or a less common cause of stroke or TIA.	Imaging supports the clinical diagnosis with no suggestion of an alternative aetiology. No mimic diagnosis suggested by other investigations	Full (as appropriate)
**Definite mimic**	Positive clinical evidence from history/examination and/or previous medical history consistent with a mimic condition	Evidence consistent with a mimic diagnosis on CT/MRI imaging or other investigations.	No new secondary prevention.
**Probable mimic**	Symptoms more suggestive of a mimic than a vascular territory. Vascular risk factors may be present. No relevant PMH of a mimic condition	No CT or MRI evidence of new stroke but no alternative imaging diagnosis. No mimic diagnosis suggested by other investigations.	No new secondary prevention.
**Unclear**	Symptoms not typical of a vascular territory and/or time course not typical of stroke or TIA. No reason for stroke or TIA to occur. No positive clinical evidence from history/examination or previous medical history consistent with a mimic condition	No CT or MRI evidence of new stroke but no alternative imaging diagnosis. No mimic diagnosis suggested by other investigations.	Simple or no new secondary prevention considered appropriate.

Following assignment of a diagnosis, clinical and imaging data collected for the study will be reviewed by an independent clinician at the study coordinating centre to determine if the diagnosis assigned and clinical/imaging information concur. If data do concur, the assigned diagnosis will be confirmed as appropriate. If the data do not concur, the case will be discussed by a Diagnostic Adjudication Committee which will comprise a stroke specialist from the study coordinating centre team, the local hospital clinician responsible for assigning the diagnosis and another local clinician who was not involved in making the diagnosis. All are blinded to the SMARTChip assay outcome. The committee will meet by teleconference and review anonymised routine clinical information available up to day 7 or discharge if sooner, to agree a diagnosis.

In addition, irrespective of whether the local diagnosis assigned and clinical/imaging data concur or not, where ‘unclear’ or ‘probable’ stroke mimic diagnoses (categories B–J in the framework) are selected, Diagnostic Adjudication Committee review will take place. This is to check that there is reasonable evidence that these categories are appropriate as typically there will be greater reliance upon clinical judgement than objective information from any routine investigations.

For ‘unclear’ cases, if the committee cannot reach consensus (e.g. because of missing clinical information or lack of adjudicator consensus), the final diagnosis will still be listed as ‘unclear’, and these patients will not be included in the diagnostic accuracy analyses as it has not been possible to determine any diagnosis. Data for these participants will still be reported. All other diagnoses will be used in the analyses as described in the ‘Statistical analyses’ section.

#### Substudy

For the substudy, a reference standard is required for ischaemic stroke with LVO. Angiography of the cerebral circulation via CT or MR imaging conducted as part of standard clinical care will be used. Angiography is not routinely performed for all suspected stroke patients as clinical or non-contrast radiological examination can decree it unneeded. It is usually performed for patients with National Institute for Health Stroke Scale (NIHSS) [20] score of >5 presenting within 6 h of symptoms onset, where plain CT has not shown a haemorrhage or another radiological diagnosis for the acute symptoms, e.g. tumour.

For patients undergoing CT or MR angiography (CTA or MRA), LVO will be defined as present if angiography demonstrates reduced filling in any large branch of the anterior cerebral circulation as assessed using the Ten Point Clot Burden Score [21]. A score <10 will indicate the presence of LVO. All CTAs will be reported by a consultant neuroradiologist blinded to patient and study information. Local clinical routine imaging reports will also be obtained if available, but these will not alter the data to be used for the main study analyses. The frequency and nature of any discrepancies between the reports will however be reported on study completion.

For participants who do not undergo angiography because they do not meet the clinical criteria (e.g. mimic conditions, haemorrhagic stroke, mild ischaemic stroke with NIHSS < 6 and TIA) and the probability of LVO is remote, it will be assumed that they do not have LVO. However, there may also be participants with ischaemic stroke of sufficient severity to be potentially caused by LVO (i.e. NIHSS >5), but who do not undergo angiography. This is usually because of an early clinical judgement that they would not be offered MT (e.g. due a low Alberta Stroke Program Early CT Score [22] or significant comorbidities) or because MT is not available. For these participants, it will not be possible to make a confident assumption about LVO status, and therefore, their data will not be included in diagnostic accuracy analyses concerning LVO. Available data for these participants will still be reported.

### Study data collection

For participants who give consent for enrolment in this study, SMARTChip WBPC assay data, clinical diagnosis data, imaging data and routine healthcare data to confirm study eligibility and conduct study analyses will be collected. WBPC assay data will be recorded by the SMARTChip reader and subsequently harvested to be linked with clinical data. The SMARTChip assay process, imaging data, clinical diagnosis data and other routine healthcare data will be recorded onto study-specific case record forms (CRFs) by ambulance personnel, NHS research support staff or other hospital clinical staff trained to deliver this research project.

Data from CRFs will be entered locally onto a secure online database. Patients will be identified by a unique study number only (for ambulance-tested patients, the ambulance used SMARTChip ID number; for hospital only tested patients where calibration/buffer of more than one SMARTChip is permitted, the ID number of the SMARTChip which had the blood sample applied will be used). Where consent is not obtained, non-identifiable data about the assay attempt only will be added to the study database to allow the total number of assay attempts to be reported.

#### Data related to pre-admission, collected if an ambulance reading attempt was undertaken, recorded by ambulance staff

##### Data about ambulance SMARTChip assay

SMARTChip ID number (will be provided on sticky labels to add to study forms to avoid transcription errors), date and time of completion of the SMARTChip assay, any complications from fingerprick sampling (no/yes: free text), any reason for the SMARTChip assay process to be aborted before completion (no/yes: free text)

##### Data for confirmation of inclusion and exclusion criteria

Age, symptom onset or last known to be well date and time (ambulance personnel judgement), symptoms which suggest stroke (face weakness, arm weakness, speech disturbance, leg weakness, visual loss, eye deviation, double vision, other), first recorded conscious level (AVPU scale), first recorded capillary blood glucose reading, any external signs of acute trauma (yes/no), received chemotherapy or radiotherapy for cancer within the last 7 days (yes/no), hospital conveying to

##### Other data related to pre-admission

Professional who suspected stroke (technician, paramedic, other ambulance role), first systolic blood pressure reading, first heart rate reading, first peripheral oxygen saturation reading, aural temperature reading, possible blackout today (yes/no/unknown), possible seizure today (yes/no/unknown), current headache (yes/no/unable to respond), previous medical history of epilepsy (yes/no/unknown), previous medical history of migraine (yes/no/unknown), date and times of 999 call; ambulance on scene and hospital arrival.

#### Data related to a hospital reading attempt and recorded by hospital staff

Hospital SMARTChip ID number(s) (will be provided on sticky labels to add to study forms to avoid transcription errors), date and time of completion of the hospital SMARTChip assay, complications from fingerprick sampling for hospital SMARTChip assay (no/yes: free text), any reason for the hospital SMARTChip assay process to be aborted before completion (no/yes: free text)

#### Data related to day 1 of admission, collected if either an ambulance and/or a hospital reading attempt was undertaken and consent is obtained, recorded by hospital staff

Demographic information (age, gender), date and time of hospital admission, first recorded conscious level on admission (AVPU scale), first blood pressure reading on admission, first heart rate reading on admission, first temperature reading on admission, first peripheral oxygen saturation on admission, first blood glucose reading on admission (capillary or serum glucose), any external signs of acute trauma noted on first clinical examination (yes/no), symptom onset or last known to be well date and time (hospital judgement), previous vascular history (stroke, TIA, heart failure, atrial fibrillation, diabetes, hypertension), previous neurological history (migraine, seizures, any diagnosis of dementia), recent significant trauma history within the preceding 7 days (surgery, fractures, wounds), recent inflammation history within the preceding 7 days (infections requiring new antibiotic treatment, intravenous chemotherapy treatment or radiotherapy received, acute exacerbation of a musculoskeletal condition, e.g. gout), current medication history (dipyridamole, anticoagulants, allopurinol), usual level of mobility (independent, independent with walking aid, physical assistance, cannot walk), standard laboratory bloods on admission (renal function: creatinine, urea, sodium, potassium; glucose; C-reactive protein; full blood count: haemoglobin, leucocytes, platelets), hospital admission (yes/no, discharged/no, transferred directly to another hospital/no, died in ED), date and time left ED, first ward if admitted locally (stroke unit/medical admissions ward/other medical ward/trauma ward/surgical ward/other), destination ward if transferred directly to another hospital (neurosurgical/stroke unit/trauma/other), stroke symptom severity on admission (National Institute of Health Stroke Score [20] -this is routinely documented for most suspected stroke patients, but if this is found to be missing from routine records, it will be completed by the NHS research team from direct assessment of the patient or using the clinical examination documented in routine records), most likely clinical stroke subtype according to new symptoms (Oxford Community Stroke Project classification [23]), intravenous thrombolysis treatment administered (yes/no), date and time of bolus administration (if thrombolysis received), mechanical thrombectomy treatment administered (yes/no), date and time of arterial puncture (if thrombectomy received), if thrombolysis or thrombectomy were received, NIHSS recorded at 24–48 h after treatment

##### If a hospital only assay reading attempt was undertaken

Symptoms which suggested stroke (face weakness, arm weakness, speech disturbance, leg weakness, visual loss, eye deviation, double vision, other), professional who suspected stroke at hospital (ED nurse, ED junior doctor, ED senior doctor, stroke nurse, stroke junior doctor, stroke senior doctor)

##### If an ambulance assay reading was attempted but a hospital assay (for the sub-study) was not

Reason why hospital WBPC assay was not attempted (patient >6h since symptom onset/admitted out of hours/trained assay user not available/reader malfunction/no SMARTChip available/other)

#### Data related to day 7 of admission (or death/discharge if this is sooner than day 7) collected if either an ambulance and/or hospital reading attempt was undertaken and consent is obtained, recorded by hospital staff

Deceased, inpatient or discharged alive at day 7, if deceased, cause of death according to death certificate, if discharged, discharge date, confirmation that symptom onset date/time recorded on day 1 is still correct at day 7 or death/discharge (no change/change. If changed: new date/time), length of stay on the stroke unit (0–7 days), COVID-19 status (if available), primary clinical diagnosis for this attendance in place at day 7 as documented in the medical records (free text), primary clinical diagnosis in place at day 7 according to a pre-defined framework, description of the clinical rationale on which the primary clinical diagnosis (according to the pre-defined framework) was selected (free text to describe clinical features including vascular risk factors and investigations), for all participants, an anonymised copy of the discharge letter will also be requested. This will be used during independent adjudication of reference standard diagnoses.

#### Imaging data collected if either an ambulance and/or hospital reading attempt was undertaken and consent is obtained, recorded by hospital staff

Brain imaging performed (yes/no), brain imaging date(s), time(s) and modality (CT/MR/CTA/MRA/CTP) performed during the first 7 days of admission (or death/discharge if sooner), brain imaging result(s) free text (formal reports/entries in the medical records if formal reports are unavailable), CT or MRI angiography performed on day 1 (yes/no), CT perfusion imaging performed on day 1 (yes/no), if perfusion imaging performed (according to the local radiological software output): core volume (CV: ml), penumbra volume (PV: ml), cerebral blood volume (CBV: ml), time to peak (TTP: seconds), mean transit time (MTT: seconds), cerebral blood flow (CBF: ml/s)

For participants whose data will be included in the sub study (i.e. those where a hospital SMARTChip assay followed an ambulance assay), anonymised CT, CTA, MR and MRA images performed in the acute phase (i.e. < 12 h since symptom onset) will also undergo separate blinded neuroradiologist review using a checklist to record: changes of cerebral ischaemia, Alberta Stroke Program Early CT Score [22], other pathological findings (e.g. subdural haematomas, tumours, subarachnoid blood), Ten Point Clot Burden Score [21], and Extended Thrombolysis in Cerebral Infarction scale [24].

### Blinding

Patients, clinicians and research support staff will be blinded to WBPC results. Clinicians responsible for adjudication and neuroradiologists responsible for providing the independent imaging reports will also be blinded to WBPC results. Checks on raw WBPC data conducted by Sarissa Biomedical staff will be without access to any clinical data.

### Staff training and awareness

Study-specific training will be provided for ambulance personnel, stroke teams and research support staff in sessions which will explain the study objectives and demonstrate use of the SMARTChip and Reader and completion of the study documentation and database as appropriate.

### Study withdrawal

No specific withdrawal criteria have been pre-set. Participants may withdraw from the study at any time for any reason. Data collected prior to withdrawal will be used in the study analysis unless the patient or their representative requests that this should not be the case. Should a decision to withdraw from the study be made, a reason for withdrawal will be sought, but participants can choose to withdraw without providing an explanation.

### Safety evaluation

This is a clinician-blinded observational study of a new diagnostic technology which will not change patient treatment. The fingerprick sampling procedure for the WBPC assay is already performed by ambulance and hospital staff during routine measurement of capillary blood glucose concentrations on all suspected stroke patients. The procedure for capillary sampling for the WBPC assay is identical to this routine clinical practice. Each SMARTChip is single use only. There will be no direct contact between patients and the portable reader device, which itself does not contain any biological or hazardous materials. The risks from participation should be no greater than standard clinical care, and there were no safety issues reported in previous studies.

Study data collection will include documentation of any complications following the blood-sampling procedure or use of the SMARTChip reader device.

Should a medical event occur which is serious (results in death, is life-threatening, requires inpatient hospitalisation or prolongation of existing hospitalisation, results in persistent or significant disability or incapacity, consists of a congenital anomaly or birth defect, otherwise considered significant by investigator) and is perceived to be related to the use of the SMARTChip assay, a separate study Serious Adverse Event form will be completed. All such events will be considered ‘unexpected’ and reported to the chief investigator, study sponsor and Research Ethics Committee.

### Sample size

1. Pilot study. There is no pre-specified sample size for the hospital pilot study. This phase will continue until agreement is reached about technical performance parameters as described below.
2. Hospital cohort study. In the hospital setting, we consider that test specificity for mimics is more important than sensitivity such that mimics are directed away from the emergency stroke pathway whilst minimising removal of stroke patients in error. To detect a 90% (lower 95% confidence limit, 80%) diagnostic specificity for mimic identification assuming a mimic rate of 25% (i.e. 75% non-mimic or true stroke patients), one-tailed 5% type I error rate and 90% power, 167 participants (125 non-mimics) are required for the ‘per-protocol’ analysis. For validation of the hospital statistical model (combined clinical data and SMARTChip assay reading), 100 further non-mimics are needed, i.e. a further 134 patients in total. This gives a sample size of 301 (225 non-mimics). However, this target anticipates a non-mimic rate of 75% in the test population. If the mimic rate is higher, additional patients will be required. Inflating for 20% for participants who cannot feature in the ‘per-protocol’ analysis (see definition below in ‘Main hospital and ambulance study analyses’) gives an initial target sample size of 377 participants (including 281 non-mimics).
3. Ambulance cohort study. In the pre-hospital setting, we consider that test sensitivity for mimics is more important than specificity to maximise removal of mimics from the emergency stroke pathway for service efficiency. To detect an 88% (lower 95% confidence limit, 80%) diagnostic sensitivity for mimic identification assuming a mimic rate of 40%, one-tailed 5% type I error rate and 90% power, 498 study participants (199 mimics) are required for the ‘per-protocol’ analysis (Agresti-Coull method [25]). For validation of the statistical model (combined pre-clinical data and SMARTChip assay reading), 100 further mimics are needed [26], i.e. a further 250 patients in total. This gives a sample size of 748 (299 mimics). However, this target reflects a typical FAST mimic rate of 40% amongst suspected stroke admissions. If the mimic rate is lower, additional patients will be required. Inflating for 20% of participants who cannot feature in the ‘per-protocol’ analysis (see definition below in ‘Main hospital and ambulance study analyses’) gives an initial target sample size of 935 patients (including 374 mimics).

In both the hospital and ambulance cohorts, the proportion of patients who are not eligible for inclusion in the per-protocol analysis will be monitored prospectively, and the sample size targets increased or decreased as required.

### Pilot study analysis

The technology supporting the SMARTChip WBPC assay has undergone modification for use in this study. Prior to embarking on a diagnostic performance evaluation, it is necessary to confirm that key technical aspects are functioning as expected.

The following will be monitored although this is not an exhaustive list and other issues may arise that will require review:

- Chip calibration failure rate
- Blood measurement failure rate
- Reader software malfunction
- The range of WBPC readings being obtained is consistent with an expected range for suspected stroke from previous studies. The range will be monitored without access to clinical data.

This pilot phase will not be time or sample size limited but driven by accruing data which will determine whether any action needs to be taken. The technology and/or user training may need to be revised in an iterative way necessitating pause in patient testing whilst this is achieved. If any technology change would result in a change to the patient experience, the protocol and/or patient facing information will be amended accordingly and submitted for reapproval prior to resuming testing.

To facilitate any investigations, it may be necessary to share some clinical data collected (e.g. unblinded reference standard data) with Sarissa Biomedical and/or study investigators. Only non-identifiable information will be included.

The pilot phase will be considered completed and the study to have entered phase 2 (main hospital cohort part 1) once Sarissa Biomedical and the study investigator team are satisfied that accruing data indicates that the technology is functioning as expected. Data collected in the pilot phase which results in a pause to testing and alterations to the technology and/or training will not be used in any diagnostic performance evaluations (i.e. phase 2 onwards).

A record will be kept of all alterations made to the technology during the pilot phase.

### Main hospital and ambulance study analyses

All statistical analysis will follow quality assurance processes including taking account of relevant reporting guidelines such as STARD (Standards for the Reporting of Diagnostic Accuracy Studies) 2015 [27].

#### Visual data exploration

The data (i.e. WBPC measurements and clinical data) will be explored with visualization tools such as histograms, box and whisker plots and scattergrams to assess distribution patterns, detect missing data and outliers and look for associations and interactions between variables.

#### Analytical data exploration

Data will be explored with analytic tools. Univariate analysis will be used to investigate the linearity of the relationships between the dependent and independent variable(s). If relationship(s) are not linear, the log transformation and squared transformation will be attempted. If a transformation significantly lowers the Akaike Information Criterion (AIC), then, the variable will be transformed for use in the model selection step.

#### Analysis populations

There will be two analysis populations:

- A per-protocol (PP) group will only include participants who have a verified WBPC reading, a reference standard diagnosis (i.e. not ‘unclear’) and who met the inclusion and exclusion criteria. In the emergency pre-hospital or hospital setting, it is not uncommon for initial assessment information to be later considered inaccurate as further details emerge. Study data recorded about eligibility information will be reviewed, and any tested patients who did not meet the eligibility criteria will not be included in the PP analysis. In addition, for ambulance-tested patients, data separately recorded at the hospital will be considered more accurate for age, symptom onset time and receipt of chemotherapy or radiology, and these data will be used instead of the ambulance-recorded data to determine eligibility for the PP group for these criteria.
- An intention-to-test (ITT) group will include participants who did and did not meet the eligibility criteria and who had either a verified or an unverified WBPC, and a reference standard diagnosis (‘unclear’ will not be included).

#### Data not contributing to analysis populations

For participants where it is not possible to assign a reference standard diagnosis (i.e. unclear), available data will be reported but will not contribute to diagnostic accuracy analyses.

Where the WBPC assay technology fails prior to the point of fingerprick sampling (i.e. calibration or buffer failure), only limited data about the test will be recorded. These data will be reported.

#### Statistical analyses

1. Objective 1: to determine the diagnostic accuracy of SMARTChip assay WBPC readings for identification of stroke mimic conditions when a reading is obtained in the pre-hospital setting, i.e. the test is conducted on patients suspected to have stroke by ambulance staff

For the PP group, logistic regression analyses with binary diagnosis of mimic or stroke (with TIA grouped with stroke as the intended purpose of the test is to identify mimic) as the outcome variable and WBPC reading as the explanatory variable will be used to construct a receiver operating curve (ROC) for all possible test thresholds. Area under the ROC curve and optimal sensitivity, specificity, and negative and positive predictive values, will be reported with 95% confidence intervals (CI). Optimal thresholds for sensitivity and specificity will be chosen. As we consider that test sensitivity is more important than specificity in the ambulance, the optimal threshold will be chosen to maximise sensitivity for mimics, minimum 80%, but aiming to keep estimated specificity for mimics above 70%. At lower levels of sensitivity and specificity, the test is unlikely to be considered of value.

For the ITT group, sensitivity and specificity will be calculated using a two-by-two table. Patients with a verified WBPC reading will be assigned a ‘test diagnosis’ according to the optimal threshold established in the PP analysis, and patients with an unverified WBPC will be assigned a test diagnosis of stroke. If the SMARTChip was deployed in clinical practice and an unverified reading was obtained, patients would continue to be managed as suspected stroke as there would be no extra information to exclude this possibility.

2. Objective 2: to determine the diagnostic accuracy of SMARTChip assay WBPC readings for identification of stroke mimic conditions when a reading is obtained in hospital, i.e. the test is conducted on patients suspected to have stroke by hospital staff and when an ambulance test has not been undertaken

For this objective, analyses as described above for objective 1 will be conducted. In terms of optimal thresholds, as we consider that test specificity is more important than sensitivity in hospital, the optimal threshold will be chosen to maximise specificity for mimics, minimum 80%, but aiming to keep estimated sensitivity for mimics above 70%. At lower levels of specificity and sensitivity, the test is unlikely to be considered of value.

3. Objective 3: to develop pre-hospital and hospital statistical models which combine routinely available clinical data with SMARTChip assay WBPC readings to predict a stroke mimic diagnosis

Key variables will be added to the PP models described above to determine if this could significantly increase their accuracy. Clinical variables that would be available to ambulance/hospital staff will be included to reflect those available at the point of testing. Stepwise regression with backward elimination will be used to select the clinical covariates that most influence the diagnosis of mimics in conjunction with the WBPC readings.

A stepwise variable selection procedure will be used, and only variables that significantly improve the AIC will be retained in the model. Sensitivity, specificity, ROC AUC (with confidence intervals) and threshold will be reported if a suitable model is found.

4. Objective 4: to prospectively determine the diagnostic accuracy of the statistical models from objective 3

The models derived under objective 3 will be used to predict mimic/stroke status, and sensitivity, specificity and negative and positive predictive values will be reported with 95% confidence intervals (CI) for the PP population.

5. Objective 5: to report the failure rate of the SMARTChip assay when used in the pre-hospital and hospital settings

Reasons why a SMARTChip assay measurement was attempted but not obtained will be categorised and reported. This will include the following:

- Failed SMARTChip calibration or buffer
- Failed SMARTChip reading following successful calibration (i.e. unverified reading)
- User-reported clinical or operational reason for aborting the calibration or reading process

A failure rate will be calculated for pre-hospital and hospital settings.

6. Subgroup and exploratory analyses

Diagnosis accuracy calculations will be performed on a pre-specified subgroup of the PP population consisting of only those patients with a reference standard diagnosis of ‘Definite Stroke’ and ‘Definite Mimic’ (this subgroup analysis is being undertaken as it considers the highest level of clinical confidence in the diagnosis and therefore allows exploration of the SMARTChip assay performance under ideal conditions). Contingent on the results of the study, it may be important to carry out some data-driven exploratory analyses. These will be determined post hoc and reported as such.

7. Substudy analysis
  - Sub-study objective 1: to explore the diagnostic accuracy of two sequential SMARTChip assay WBPC readings for identification of large vessel occlusion stroke using a reading obtained in the pre-hospital setting and a second reading obtained in the hospital setting

This analysis will include only patients who have both a pre-hospital- and hospital-verified WBPC reading, a reference standard assigned and who meet all the eligibility criteria for both tests.

Logistic regression analyses with diagnosis (LVO) as the outcome, hospital WBPC reading as the explanatory variable and pre-hospital WBPC reading as a covariate will be used to construct a receiver operating curve (ROC) for all possible test thresholds. Area under the ROC curve and optimal sensitivity, specificity, and negative and positive predictive values will be reported with 95% confidence intervals (CI). As for the main in-hospital study, the optimal threshold will be chosen to maximise specificity.

b) Sub-study objective 2: to develop and retrospectively explore the diagnostic accuracy of a statistical model which combines routinely available clinical data with pre-hospital- and hospital-obtained SMARTChip WBPC readings to predict the presence of large vessel occlusion.

Key variables will be added to the model described above to determine if this could significantly increase its accuracy. Clinical variables that would be available to ambulance and/or hospital staff will be included to reflect those available at the point of testing. Multivariate logistic regression will be conducted, with diagnosis (LVO) as the outcome and pre-hospital and hospital WBPC readings along with variables considered to be of possible clinical importance (such as blood pressure, pre-hospital FAST symptoms, NIHSS) as the explanatory variables. A stepwise variable selection procedure will be used, and only variables that significantly improve the ROC AUC will be retained in the model. Sensitivity, specificity, ROC AUC (with confidence intervals) and threshold will be reported if a suitable model is found. As for the main in-hospital study, the optimal threshold will be chosen to maximise specificity.

c) Substudy subgroup and exploratory analyses

Contingent on the results of the study, it may be important to carry out some data-driven subgroup or exploratory analysis. These will be determined post hoc and reported as such.

### Study monitoring, quality control and quality assurance

The chief investigator will have overall responsibility for study conduct. The local principal investigators will be responsible for the day-to-day study conduct at their individual NHS sites. The study will be managed by a coordinating centre based at Newcastle University who will provide training and day-to-day support for the sites. Quality control will be maintained through adherence to the Newcastle Biomedicine Clinical Research Platform standard operating procedures, the study protocol and research governance regulations. The study may be subject to inspection and audit by the Northumbria Healthcare NHS Foundation Trust under their remit as sponsor. A Study Steering Committee will be convened to provide oversight of the trial. This will comprise of the study investigators plus an independent member. This committee will aim to meet 6 monthly.

### Dissemination of results

The study will be presented at national and international conferences and reported in peer-reviewed journals. Reports will be written for the study funder, sponsor and regulatory bodies. A lay summary of the results will be available for study participants.

## Discussion

Early diagnostic uncertainty about the cause of suspected stroke symptoms results in displacement of non-stroke mimic patients from more appropriate services, inappropriate demands on specialist resources and delayed access to specialist care and time-critical reperfusion therapies for stroke patients. Blood biomarkers have not previously been shown to be useful in emergency stroke assessment due to delayed elevation and a need for complex assays [13–15]. In addition, markers of inflammatory response or vascular risk have not improved upon clinical assessment alone [13, 28]. However, there is now evidence to suggest that blood purine concentration which rises rapidly during hypoxic tissue injury may be able to assist with urgent differentiation of stroke from mimic conditions [16–19].

This study will determine the performance of a portable point of care fingerprick measurement of blood purine concentration for the identification mimic patients within the suspected stroke population. We will also consider whether blood purine readings show greater diagnostic accuracy when combined with other information available to clinicians such as symptom severity. A substudy will explore whether serial purine readings could be an early indicator of large vessel occlusion which could be an alternative use of the test.

If test performance to identify non-stroke mimic conditions is satisfactory, future deployment in ambulances and emergency departments could assist with urgent triage of patients with suspected stroke symptoms and enable more appropriate direction of patients to stroke or non-stroke services. Improved service access could consequently result in better outcomes through faster access to appropriate treatments.

## Study status

At the time of submission of this manuscript, recruitment to the hospital cohort is in progress. Protocol version 4 dated 14 July 2020 was used to prepare this manuscript.

## Data Availability

Not applicable

## Abbreviations

AVPU: Alert, Voice, Pain, Unresponsive
CRF: Case record form
CT: Computerised tomography
CTA: CT angiogram
CTP: CT perfusion
FAST: Face Arm Speech Time
HASU: Hyperacute stroke unit
LVO: Large vessel occlusion
MRA: MR angiography
MRI: Magnetic resonance imaging
MT: Mechanical thrombectomy
NHS: National Health Service
NIHSS: National Institute of Health Stroke Scale
NNT: Number needed to treat
PI: Principal investigator
ROC: Receiving operating curve
STARD: Standards for the Reporting of Diagnostic Accuracy Studies
TIA: Transient ischemic attack
WBPC: Whole blood purine concentration
